# The Electronic Bee Spy: Eavesdropping on Honeybee Communication *via* Electrostatic Field Recordings

**DOI:** 10.3389/fnbeh.2021.647224

**Published:** 2021-04-28

**Authors:** Benjamin H. Paffhausen, Julian Petrasch, Uwe Greggers, Aron Duer, Zhengwei Wang, Simon Menzel, Peter Stieber, Karén Haink, Morgan Geldenhuys, Jana Čavojská, Timo A. Stein, Sophia Wutke, Anja Voigt, Josephine Coburn, Randolf Menzel

**Affiliations:** ^1^Department Biology, Neurobiology, Freie Universität Berlin, Berlin, Germany; ^2^Department Information Science, Freie Universität Berlin, Berlin, Germany; ^3^CAS Key Laboratory of Tropical Forest Ecology, Xishuangbanna Tropical Botanical Garden, Chinese Academy of Sciences, Kunming, China; ^4^Independent Researcher, HAPE Imkerei GmbH, Neulußheim, Germany; ^5^Complex and Distributed IT Systems, Technische Universtät Berlin, Berlin, Germany; ^6^Department of Physics, University of Oslo, Oslo, Norway

**Keywords:** honey bee (*Apis mellifera L.*), social, electrostatic field, behavior, datalogger

## Abstract

As a canary in a coalmine warns of dwindling breathable air, the honeybee can indicate the health of an ecosystem. Honeybees are the most important pollinators of fruit-bearing flowers, and share similar ecological niches with many other pollinators; therefore, the health of a honeybee colony can reflect the conditions of a whole ecosystem. The health of a colony may be mirrored in social signals that bees exchange during their sophisticated body movements such as the waggle dance. To observe these changes, we developed an automatic system that records and quantifies social signals under normal beekeeping conditions. Here, we describe the system and report representative cases of normal social behavior in honeybees. Our approach utilizes the fact that honeybee bodies are electrically charged by friction during flight and inside the colony, and thus they emanate characteristic electrostatic fields when they move their bodies. These signals, together with physical measurements inside and outside the colony (temperature, humidity, weight of the hive, and activity at the hive entrance) will allow quantification of normal and detrimental conditions of the whole colony. The information provided instructs how to setup the recording device, how to install it in a normal bee colony, and how to interpret its data.

## Introduction

A honeybee colony is a well-organized unit of social life that is composed of highly interacting groups of single organisms with different duties, age-dependent behavioral routines, and experience. Many of the messages communicated between these organisms are accessible by electric-field measurements. These are electrostatic signals that workers produce due to their body movements, e.g., their dances, shivering to control temperature, fanning behavior to regulate hive humidity and CO_2_, “stop” and “whooping” signals, and overall motor activities characteristic of arousal states, preparation for play flights of young bees, and preparation for swarming. Dances are particularly rich in information because they encode specific meaning in a symbolic form (von Frisch, 1967). Rhythmic movements of bee bodies, in whole or in part (e.g., the abdomen and the wings), are technically easily observable because they produce characteristic patterns of electrostatic fields (ESF; Greggers et al., 2013). This is because the wax-covered body surface of bees charge up electrostatically due to friction between body parts, between animals inside of the crowded hive, and between the air and body during flight. We used these signals to characterize and quantify the information flow inside the hive. Here we describe how we measured the relevant signals under undisturbed bee keeping conditions, how we related these electrostatic signals to body movements of single bees, and how we shall use these signals to identify biologically meaningful states of the colony.

ESF data collected from whole colonies are relevant in many respects. Honeybees are the most efficient pollinators of economically and environmentally highly relevant plants (Klein et al., 2007; Potts et al., 2016). Dance activities tightly mirror foraging activities across an area of some 5 km radius around a hive (von Frisch, 1967). Since a dance codes the outbound component of a flight to the pollinated flowers or other food sources, available information potentially allows spatial tracking of pollination activities (Steffan-Dewenter and Kuhn, 2003; Seeley, 2011; Couvillon et al., 2014). These efforts, however, provided rather limited information because of the experimental challenges to decode the large number of dances necessary for ecological studies. ESF measurements allow for a quantification of colony-related pollination activity and its dynamics over time and space. The health of honeybee colonies, and thus their pollination efficiency depends on multiple components including season, environmental conditions, bee keeping activities, infections by parasites (viruses, bacteria, fungi, mites), and exposure of/to insecticides (Chauzat et al., 2010; Moritz et al., 2010; Morawetz et al., 2019). The latter conditions are particularly relevant in modern agricultures since many insecticides (e.g., neonicotinoids) act directly on the nervous system of honeybees (Eiri and Nieh, 2012; Casida and Durkin, 2013), and have been found to compromise not only foraging activity and navigation, but also dance communication (Van der Sluijs et al., 2015; Tison et al., 2020). Other insect pollinators (butterflies, beetles, flies, solitary bee) are also affected by insecticides, and thus monitoring the effect of insecticides on honeybee communication may provide information beyond honeybee pollination activities (Pisa et al., 2015). In this sense, ESF measurements in honeybee colonies offer access to biologically and environmentally relevant data about the health condition of ecosystems.

Our approach aims to implement a robust ESF measuring device that allows normal bee keeping activities and data collection by beekeepers without sophisticated knowledge of electronics or big-data management. The methods applied are based on the discoveries by Greggers et al. (2013), which require sophisticated laboratory instrumentation and are, thus, not suited for typical bee-keeping activities. We found that ESF signals tightly mirror biologically relevant conditions and will allow unsupervised long-term monitoring of health conditions in honeybee colonies.

## Materials and Methods

### Hive

**Figure 1:**
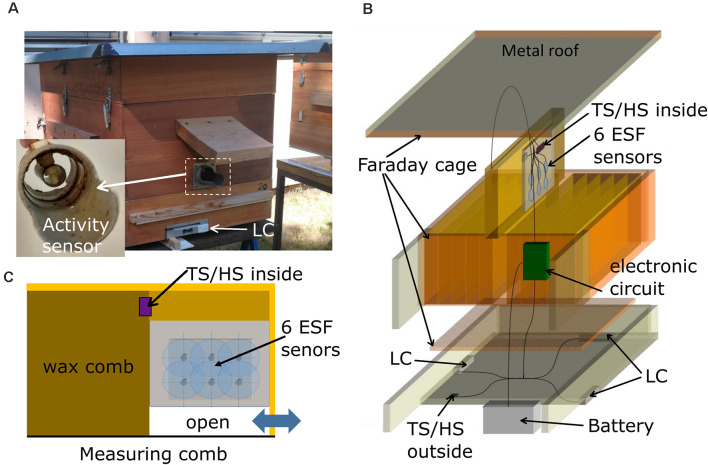
Hive construction and measuring devices. **(A)** Front and side view of the hive. A plastic tube containing a GPS receiver was fixed to a sidewall (not shown). The weight of the hive was measured with three load cells (LC). The round entrance tube was equipped with a capacity sensor of the bee traffic (insert to **A**). A sensor for external temperature and humidity was located below the extension of the hive box (arrow TS/HS outside). **(B)** The hive was built as a Faraday cage with a metal mesh between two tightly attached wooden plates, a metal mesh as the ground floor and a metal plate as the roof. The middle comb close to the entrance [blue double pointing arrow in **(C)**] contained the six ESF sensors. **(C)** Side view of the measuring comb with the six electrostatic field (ESF) sensors.

The bee hive contained 11 regular comb frames (Zander system, Holtermann, Germany) and one frame for the measuring devices (Figure 1A). The four vertical walls of the hive box were made of two layers of wood glued together with a metal mesh between them to act as a Faraday cage. The mesh layers were connected to the ground. The floor consisted of a metal mesh for ventilation and access of bees to the combs. In order to close the Faraday cage, a roof made of tin was also connected to the mesh layers and ground. The system was designed such that normal beekeeping was combined with electric-noise shielding. The back of the hive box was elongated to house the electronics and a car battery as power supply.

### Sensors

A plastic tube extending from the side-wall of the hive contained a GPS module (UBX-G7020, u-blox, China, not shown), which was used to synchronize time and spatial date information from satellites. The hive weight was monitored with three load cells (LC; 50 kg, AUTODA, China) connected to load cell amplifiers (Avia Semiconductor, HX711 ADC, China). A sensor for external temperature and humidity (HTU21D-F breakout board, Adafruit, NY, USA) was located below the extension of the hive (Figure 1A, TS/HS sensor outside). A second sensor for internal temperature and humidity was located in the measuring frame (Figure 1B, TS/HS sensor inside). The entrance consisted of a plastic tube (diameter: 50 mm, length: 10 cm) with a landing platform (Figure 1A, insert). The inside of the tube was equipped with two metal rings (width: 1 cm) that were connected to a capacity sensor (FDC1004, Texas Instruments, TX, USA). A brass tube (diameter: 1 cm) was placed in the middle of the plastic tube and served as a reference potential by being connected to ground. These capacity sensors at the entrance served as an activity measure of bees entering and leaving the hive. Changes of capacitance depended on the numbers and frequencies of bees traveling through the tube since the dielectric constant of the bee body (mainly water) is approximately 80 times higher than that of air. The high dynamic range of the sensor allowed nearly single-bee resolution, and measurements from an absence of bees to full bee beard and above.

**Figure 2:**
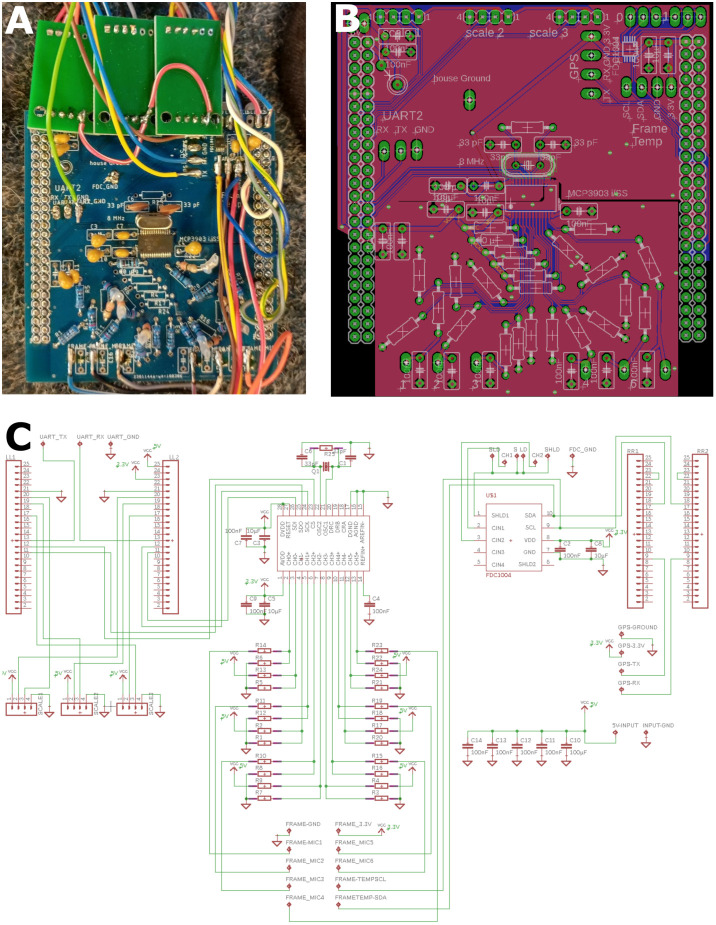
Central recording device. Inputs (ESF probes, Temperature, Humidity, Scale, GPS and entrance activity; Figure 1) were connected to the central board as well as to a SD card writer to save all data. The custom-made board was plugged onto a STM32 developer board. **(A)** Photograph of one unit. Large pin rows left and right connected to the STM32 board below. The green printed circuit boards (PCBs) on the bottom house the scale instrumental amplifiers and associated ADCs (analog-digital converters). The central SMD chip (MCP3903, Microchip, AZ, USA) digitized the analog ESF signals (middle of PCB). **(B)** PCB schematic diagram of the board in **(A)**. Note the split ground plane along the horizontal middle axis. The lower segregated ground plan isolated the upper digital traces from the sensitive analog ESF signals. **(C)** Circuit diagram of **(A)** and **(B)**.

The ESF measurements sensors were placed in a box—referred to as measuring comb—(Figure 1C) made of 4 mm thick Plexiglas sheets which occupied a third of a standard Zander frame with a thickness identical to regular combs. The sensors pointed towards the neighboring comb in the region of the dance ground close to the entrance (see double pointing arrow in Figure 1C). The back of the measuring box was shielded by a grounded metal mesh in order to prevent pickup of ESF signals from comb on the other side. An open space of 2 cm in the lower part of the box allowed bees to cross to other combs. The six ESF sensors (CJMCU-9812 MAX9812L, CJMCU, China; capacitive microphones with microphone preamplifier board) were arranged in the lower part of the box (Figure 1C). The microphone capsules were opened and their dielectric membranes were removed, eliminating any sound pressure effects and exposing the gate pin of the central J-FET. The cut-off frequency was reduced to 5 Hz by replacing the original capacitor at the entrance gate with a jumper wire. The six altered sensors were arranged in two rows with three sensors each, all facing the same direction towards the dance floor of the opposing comb. The amplified and filtered (5–20,000 Hz) analog signals are sent to a synchronous six channel delta-sigma analog digital converter (MCP3903, Microchip, AZ, USA) on a custom-designed printed circuit board (PCB) in the hive back (Figure 2).

### Central Recording Device

**Figure 3:**
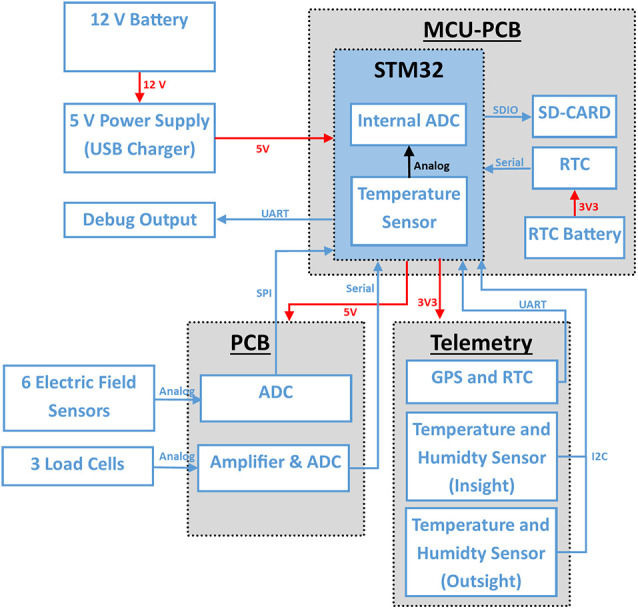
System overview: red connections show power connections and blue data lines. The system is connected to a 12 V battery. A step down boost converter generates 5 V which is also regulated by the MCU-PCB to 3.3 V for some sensors. The electric field sensors are connected to an ADC (MCP3903, Microchip, AZ, USA) on the PCB. The STM32 reads out the ADC data with a sample rate of 5,000 Hz *via* SPI in a timer triggered interrupt. The activity sensor channels are read out at 200 Hz *via* I^2^C. The scales are connected *via* a custom serial protocol and are, like the temperature and humidity sensors (I^2^C) read out every 120 s.

The board in the electronic device was a custom made 2-layer PCB (Figures 2, 3) connecting all sensors and devices to a microcontroller evaluation board (STM32F407VET, STMicroelectronics, Geneva, Switzerland) attached to it on its lower side (shield-arrangement). The custom board also housed an ADC (analog-digital converter) and related analog-signal conditioning circuits. The ADC digitized six signal channels simultaneously with 5,000 samples per second with a 24-bit resolution. The data from the six ESF sensors were saved from 06:00 to 23:59 UTC and stored on a 64 or 128 GB SD card. In addition to the ESF data, data from the temperature and humidity sensors inside and outside the hive, data from three load cells acting as a scale for the mass of the whole hive, and MCU-temperature (microcontroller-temperature), were sampled and saved every 120 s all day long. The data from both capacitance sensors at the entrance were sampled and saved at 100 samples per second from 06:00 to 23:59 UTC. Each of these three data streams were saved in binary files with regular GPS timestamps. Additionally, a unique identifier for each hive and a hardware identifier of the microcontroller was saved as well. We chose to store all data in a binary format to use the data storage on the SD cards efficiently. The whole system was powered by a generic 12 VDC, 60–100 Ah car battery, which provides electricity for more than 10 days. The battery needed to be recharged approximately once per week, at which time, the SD card was replaced by an empty one. The binary data were converted into CSV (comma separated values) files and processed by a custom program written in Python 3.

### Data Processing

The resulting CSV files were structured in the following way: one file contained the data of the six ESF channels together with the corresponding time stamps for a duration of 200 s per file, another file contained all daily data on temperature, humidity and load cells, and a third file contained the two channels of the capacitance sensors at the entrance and daily timestamps. In addition, weather data were downloaded automatically *via* the Darksky API^1^ for the hive’s location determined by the GPS sensor and matching the data’s timestamp. The weather data were used for analyses in addition to data from the temperature and humidity sensors outside the hive. Example of the data can be found in the article’s Supplementary Materials (raw data, converted data of all types, one typical summary PDF and WAV files). A summary PDF file for quick analyses was produced together with optional WAV files (see Supplementary Materials).

**Figure 4:**
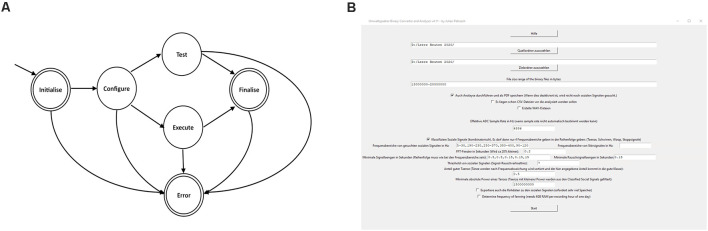
State machine and graphic surface of the converter and analyzer. **(A)** Schematic diagram of the state machine (see text). **(B)** The converter and analyzer graphical user interface.

A state machine, built with function pointers, was used to make the code more structured (Figure 4A). At the start of the monitoring unit the RTC (real time clock) was synchronized with the GPS time (if no GPS time was available after waiting 5 min it went into an error-state and rebooted), and then the load-cells were initialized. When no error occurred in the initializing-state it continued to the configuring-state. The configuration instruction was loaded, verified that it was time for a recording, total number of measurements was calculated and switched to the execution-state. If debugging was needed the system could be switched to the testing-state where the sensors may be tested. When the desired number of electric-field measurements was reached the system switched to the finalizing-state where the microprocessor was set to the standby mode in order to save power. After waking up from the standby mode it switched again to the initializing-state. If an error occurred, the system switched to the error-handling-state where an error message was printed (*via* UART) and switched again to the initializing-state.

The converter and analyzer graphical user interface (Figure 4B) created CSV-files (comma-separated values), enriched it with online weather data, detected the social signals and created a report. The user input needed for analysis was as follows: first, the user chose the folder containing the raw binary files. Next, the scope of conversion and analysis was selected, including optional steps like the generation of WAV files of the ESF traces for manual investigation in audio software. The program allowed the generation of a PDF file summarizing all results like temperatures and alike as well as dances over time in a basic human-readable format (see Supplementary Materials). The program also allowed for easy change of important parameters e.g., relevant social signal frequencies as well as minimum duration of such signals to be detected. This tool was intended for use by non-data scientists. In cases of incoherent inputs, the program prompted insightful error messages to debug the error.

### Validation

We related the recorded ESF signals to the animals behaviors in multiple ways. Greggers et al. (2013) reported a method that allowed to record simultaneously, dances by video and ESF using a transparent potassium chloride electrode. Tison et al. (2016, 2020) found correlations between waggle dances related ESF signals and uptake of neonicotinoids. In addition, the Supplementary Video shows a set-up with an observation hive together with the voice recording of an experimenter during simultaneous ESF, video recordings and visual inspection of dancing, fanning and stop signal producing bees. As shown in the video ESF were recorded *via* square-like wires that were connected to the ESF sensors. Multiple other set-ups were used to observe the behavior by eye together with a voice protocol that allowed off-line comparison with ESF recordings. As described below the ESF data stream was analyzed by the converter software to identify and label waggle-dance-related signals (WRS), short-pulse-related signals (SRS) and fanning-related signals (FRS). To verify those signals we investigated 97 files from different systems e.g., hives during different seasons and years. The classification of WRS, SRS and FRS was compared between human eye based labeling and that of converter based classification. To test the system in the field we cooperated with 29 beekeepers spred all over Germany besides our own 12 measuring devices. We supported and supervised the use of the devices for up to 5 years. Our students cared for the systems and carried out their experiments in our bee garden.

PCB plans, bill of materials, code on the STM microcontroller, wiring diagram, converter code as well as analysis code can be found in the Supplementary Files. The Supplementary Files also contain example data, raw and converted as well as a PDF summary. They are available here: https://figshare.com/articles/dataset/The_electronic_bee_spy_Eavesdropping_on_honeybee_communication_via_electrostatic_field_recordings_-_Supplemental_Data_and_Codes/13490973">https://figshare.com/articles/dataset/The_electronic_bee_spy_Eavesdropping_on_honeybee_communication_via_electrostatic_field_recordings_-_Supplemental_Data_and_Codes/13490973">https://figshare.com/articles/dataset/The_electronic_bee_spy_Eavesdropping_on_honeybee_communication_via_electrostatic_field_recordings_-_Supplemental_Data_and_Codes/13490973 and https://doi.org/10.6084/m9.figshare.13490973.v1. The Supplementary Video and its description is available here: https://figshare.com/articles/media/The_electronic_bee_spy_Eavesdropping_on_honeybee_communication_via_electrostatic_field_recordings_-_Supplemental_Video/14140634">https://figshare.com/articles/media/The_electronic_bee_spy_Eavesdropping_on_honeybee_communication_via_electrostatic_field_recordings_-_Supplemental_Video/14140634">https://figshare.com/articles/media/The_electronic_bee_spy_Eavesdropping_on_honeybee_communication_via_electrostatic_field_recordings_-_Supplemental_Video/14140634 and https://doi.org/10.6084/m9.figshare.14140634.v1.

## Results

### Collected Data

Thirty-six systems were run by 29 cooperating bee keepers and students over the last 5 years. We collected 46 TB of binary data that correspond to ~15 years’ worth of continuous EFS recordings. Some devices failed during deployment, mostly due to faulty solder connections. Meanwhile most of the devices ran over a period of 4 years. Some errors occurred within weeks, some after more than a year. Some of the beekeepers had experience with electronic devices and a PC, but most of them were naïve with respect to the devices and cared only about standard bee keeping. The date came from different country sides and about half of the systems also ran during winter time.

### ESF Signatures of Social Signals

**Figure 5:**
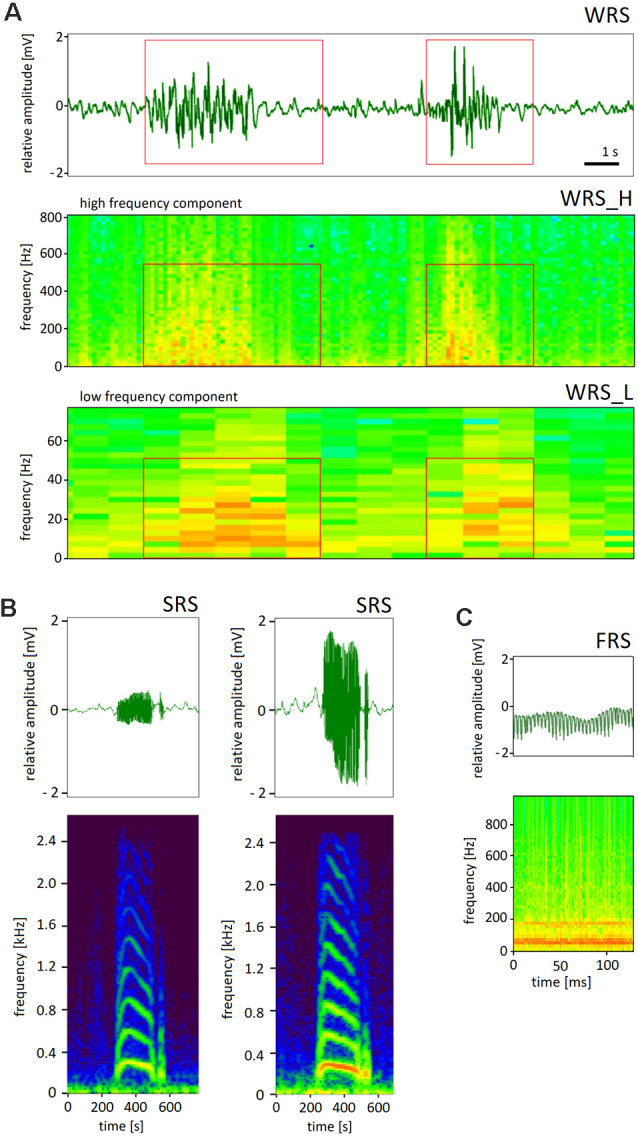
Representative examples of three classes of ESF signals. **(A)** Waggle-dance-related signals (WRS). The electrograms show the time/frequency diagram in false colors (signal power) for two frequency bands, the low frequency of the abdomen waggling (WRS_L) and the high frequency of the wing vibrations (WRS_H). **(B)** Short pulse related signals (SRS) with the time courses and corresponding electrograms for two kinds of SRS. **(C)** Fanning related signals (FRS). Note the different time scales.

The movements of the charged body of a forager bee led to patterns of ESF that were characteristic of these movements. We shall focus here on social communication signals as the most characteristic and highly stereotypical movements inside the colony. Three signals were distinguished on the basis of their characteristic frequencies and time courses, waggle-dance-related signals (WRS, Figure 5A), short-pulse-related signals (SRS, Figure 5B) and fanning-related signals (FRS, Figure 5C). WRS were composed of two frequency components, the low frequency domain (5–25 Hz, WRS_L) of the abdomen waggling, and the high frequency domain (190–230 Hz, WRS_H) of wing vibrations—partially synchronized with abdominal movements. Since the number of waggles per waggle run correlated with the distance to the indicated food source one can read the distance by counting the number of waggles and multiply them with 75 m (Haldane and Spurway, 1954). SRS last less than 1 s and were composed of high frequency signals (>350 Hz) that usually occurred in the context of dance communication. The origin and sources of these short pulses were not further characterized as these are outside the article’s scope. FRS originated from fanning behavior that led to ventilation of air inside the hive box. Fanning lasted longer than 15 s and was characterized by highly regular waves of ESF (frequency 90–120 Hz). The characteristic frequencies and temporal patterns allowed us to assign to each signal one of the three labels (WRS, FRS, SRS). The labeling process was validated by visually comparing the corresponding waveform data created by a custom-written player program in multiple example files. We found that 85% (in 97 files containing 484 dances) were classified correctly. However, the rate of missed waggle runs (false negative) was much higher: 84% (102 from 645 visually identified dance rounds). These results indicate that the number of dance rounds labeled by the converter program is highly conservative as compared to the visually labeled dance rounds.

### Environmental Parameters

**Figure 6:**
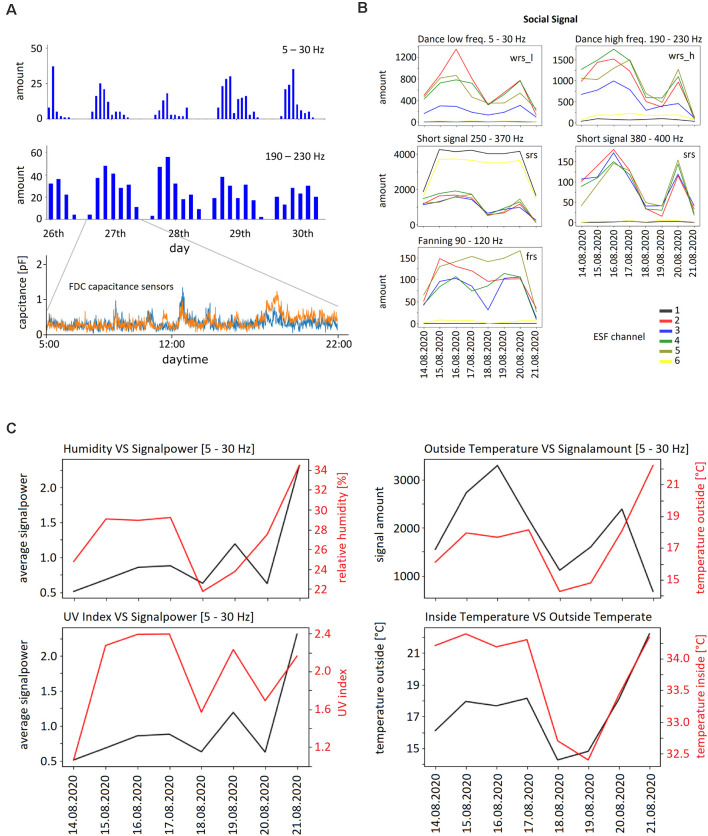
Examples of ESF signals and external as well as internal parameters. **(A)** Upper two graphs: daily rhythms of waggle dance related signals in both frequency bands (WRS_L, WRS_H) between April 20 and April 26, 2020. The bin width of the upper graph is 1 h, that of the graph below 2 h. Lower graph: signals from the two activity sensors on one day (April 17, 2020). **(B)** Examples for the five frequency bands of ESF signals recorded between August 14 and August 21, 2020 by the six ESF sensors. WRS_L: waggle dance related signal low frequency band, SRS: short pulse related signal, FRS: fanning related signal. The number of the respective signals per hour were averaged for each day. Notice the different scale of the ordinate. **(C)** Humidity outside, temperature outside, UV index, temperature inside and signal power during the time period August 14–21, 2020. Note the corresponding ordinates.

First, we examined whether the strength of the ESF depended on outdoor humidity and/or UV radiation. Such an effect would be expected if the body charge depends only on-air friction leading to higher charge in dry air and under high UV radiation. Furthermore, no ESF signals would be expected during wintertime when bees do not leave the hive. Datasets with large variation of outdoor humidity and UV radiation were compared with ESF strengths in four frequency bands, 5–30 Hz, 190–230 Hz, and 380–400 Hz. No significant correlations were found (Spearman’s correlation coefficients ranged from −0.14 to +0.18, number of data pairs *n* = 85,737 for outdoor humidity during summer periods ranging from 15% to 89% and *n* = 52,422 for UV radiation; see also Figure 6B). Next, we examined randomly selected datasets from colonies at winter time and compared them with similar numbers of randomly selected datasets from summer colonies. Significantly higher signal powers were found in all frequency bands during the summer season (*t*-test, *p* < 0.001, *n* = 1.47*10^10^) but the differences were small (1.3–1.4%).

Taken together we concluded that body charge resulted predominantly from the friction of body parts of each animal and that between the animals.

Next, we examined how social signals varied with weather conditions, seasons of the year, time of day, and internal conditions of the colony (temperature control inside the hive, brood, honey store and health conditions). Examples are shown in Figure 6. Multiple correlations were examined and will be the subject of separate analyses of the collected data.

## Discussion

Electrostatic fields (ESF) are meaningful signals to bees inside the colony. They are received *via* deflections of mechanoreceptors (antennae, possibly also mechano-sensory hairs), but not by an electric organ (Greggers et al., 2013; Clarke et al., 2017). These electrostatically induced mechanical stimuli can be learned and discriminated by bees based on their frequency components and their modulation over time. Since the frequency of waggling reaches well below the range of acoustic microphones (<30 Hz) it cannot be adequately recorded with sound-pressure microphones. The temporal resolution of our ESF recordings (5 Hz to 5 kHz) was sufficiently high to pick up characteristic frequency modulations and harmonics even for high-frequency short pulses. The short and high frequency pulses were labeled as the same category in this work although they are most likely used in different behavioral contexts and with different meaning (Bell et al., 2019). Social signals in honeybee colonies have been recorded with varying methods over the last 50 years. Most of these recordings were based on human observations or optical methods, procedures that excluded the possibility to monitor these signals under natural beekeeping conditions. Also microphone recordings relied majorly on single combs in an observation hive (Esch et al., 1965; Nieh, 1993). More natural conditions were possible in recordings of vibrational signals of the wax surface (Ramsey et al., 2017) but these measurements did not allow selective monitoring for different forms of social signals and their meaning to bees is unknown.

The current version as published in this work is fully operational, scalable, and may be adapted to various use cases. Whilst the current setup is the least complex we note that it was a time-consuming endeavor already. The high resolution of 24 bits at the ESF ADC allowed us to over-sample the signal, an approach that was of high value during the early exploration phase allowing us to analyze even minute signals. This procedure will not be necessary in the future anymore as signal amplitude and frequencies allow for lesser resolution in signal amplitude (ADC bit depth) and sampling time. The capacity sensors gathered reliable data on entrance activity with neither drift nor interfering noise being observed. These entrance capacitance data will be processed in the future in more detail (separating between arriving and departing bees, classification of massive movements) and will be related to other data either recorded (temperature and humidity inside and outside the colony) or downloaded from the weather service web site.

Classification and quantification of social signals have been performed so far offline. We are working towards implementing programs to run on the device’s MCU directly for online data reduction. Thus far, the conversion of binary data as well as the analyses of extraction and classifying the social signals did not involve machine-learning approaches, an obvious goal for the future. A further step will be to recharge the 12 V battery with a solar charging device, allowing deployment in remote areas.

Overall, the system worked successfully. The device recorded ESF signals as their related behaviors were observed. The converter software and subsequent analysis could extract the ESF signals sufficiently. However, early adaptations including those described here, contained hundreds of hand-soldered connections that caused instabilities and errors. The next generation, already in testing, is completely produced with surface-mounted components (SMD) and can be built by pick-and-place machines. This will drive the reliability to commercial standards and lower cost compared to hand soldered components. It will also allow for up-scaling the number of devices by orders of magnitude. SMD populated systems are produced fully automatically. Currently, for ease of implementation, the system does not shut down peripheral devices when they are not used. In particular, in future systems, the GPS will be switched off most of the time since it is only needed for synchronization once per day and draws considerable current while active. Furthermore, certain sensors will be switched off for periods of time leading to reduced power consumption. We have estimated that the device will run for 6 weeks with a standard lead-acid 12 V 100 Ah battery.

We found that the strength of ESF signals did not depend on UV radiation in the environment suggesting that the amount of charge in the air had only a minor or no effect on charging the bee body by friction during flight. This conclusion is supported by the finding that the strength of ESF did not correlate with the humidity outside the hive. Since, we also recorded ESF signal during wintertime we conclude that the body charge of bees resulted predominantly from friction between body parts of the same animal and between animals inside the colony.

The methods applied to identify, separate and label social signals were based on the characteristic frequency bands and the time windows over which they appeared. We confidently separated waggle-dance-related signals (WRS) from stop-signals and fanning-related signals (FRS). However, measurement-related electronic noise and, most probably, biological noise from movements of many bees in front of the ESF sensors limit the rate of correct labeling of WRS and likely caused missing signals(false negatives) and detecting wrong signals (false positives). We addressed this question by inspecting WAV files by visually evaluating both the time course of ESF recordings and the electrograms in two frequency bands as shown in Figure 5A. Initially we characterized typical WRS by observing dancing bees in an observation hive and simultaneous recording of ESF. Contrary to our expectation, false negatives were more frequent than false positives. Our expectation was based on the fact that rather similar body movements are known from other social signals like the buzzing or jostling runs of bees performed in various contexts (before a proper waggle dance is performed, arousing other bees and motivating young bees to build wax cells for food store (von Frisch, 1967; Hrncir et al., 2011). There are other forms of body movements that may emanate ESF in the frequency range of 5–30 Hz, e.g., dorso-ventral abdominal vibrations known to lead to vibrations of 10–22 Hz (Gahl, 1975), or so-called grooming dances consisting of vibrations of the entire bee body at 4–5 Hz (Land and Seeley, 2004). Furthermore, we also expected more false positives because our algorithm labeled WRS at night and during wintertime. Although it is known that bees may perform waggle dances at night (von Frisch, 1967, p351ff, personal observations) we assumed other signals than WRS may be produced by bees at times when no foraging bees are active in the environment. Thus, our current WRS labeling procedure has obvious limitations. Improvements will take additional characteristics into account. For example, the WRS_L of a single waggle run is modulated in a characteristic time course of an initial increase, then plateau followed by a decrease in frequency. In addition, waggle dances occurred mostly not as single waggle run but with bouts of waggle runs allowing improvement of the labeling of waggle dances by enlarging the time window and taking repetitive bouts of waggle run characteristic frequency bands into account. The high-frequency component of the waggle dance (WRS_H) is known to signal particularly attractive food sources (Hrncir et al., 2011) and thus may be used as indication of rich forage. Short-pulse signals (SRS) haven been recorded in bees following a dance (begging signal; Esch, 1961), stop signal (Nieh, 1993) and during swarming (Seeley, 2011). We found SRS with different frequency characteristics (basic and harmonic), durations, and frequency modulations. It has not been possible, yet, to relate these ESF signals to specific behaviors. Fanning behavior is involved in controlling temperature, humidity and CO_2_ concentrations within the colony (see below).

The recording of physical parameters both inside and outside of the colony opens up opportunities to uncover links between biological phenomena of the colony (e.g., brood cycles, preparation for swarming, health conditions) and physical parameters, a topic that will be addressed in subsequent reports. The physical parameters measured were temperature and humidity of the brood nest, the activity at the hive entrance, the weight of the hive, and the weather conditions. The controllability to regulate the brood temperature under varying external weather conditions is a highly sensitive factor of the colony’s health. For example, chalkbrood, a disease of honeybee larvae caused by the fungus *Ascosphaera apis*, can be restored if the brood temperature does not drop below 35°C (Maurizio, 1934). It has been argued that the colony responds to bacterial and viral infections by raising its temperature, also known as fever response of the colony (Seeley, 1985, p. 111). Colonies not responding with a temperature increase appear to suffer more from infections. *Nosema* infections are accompanied by an increase in humidity. Thus, combined measurements of the control of both temperature and humidity may be indicative of such infections. Exposure to insecticides compromise dance communication (Eiri and Nieh, 2012; Tison et al., 2020), individual bee navigation (Henry et al., 2012; Fischer et al., 2014; Tison et al., 2016) and learning (Tison et al., 2017). Honeybee colonies can thus serve as monitors of environmental hazards resulting from insecticide treatment in agriculture.

## Data Availability Statement

The datasets presented in this study can be found in online repositories. The names of the repository/repositories and accession number(s) can be found below: https://doi.org/10.6084/m9.figshare.14140634.v1, https://doi.org/10.6084/m9.figshare.13490973.v1.

## Author Contributions

JC wrote a script for handling and analyzing the data and edited the manuscript. JČ improved the FFT based signal detection and optimizations of the analysis and conversion. AD implemented and collected the data of the initial constructions at the electronic parts and the bee keeping boxes. MG wrote the I2C, load cell and RTC drivers and established the logic for the real time based recording. UG worked out the basic construction and provided multiple unpublished data. KH helped to build the early versions of the electronic parts and to collect data. RM provided general idea and goal of the project, literature survey, adaptations to the biology of honeybees, writing parts of the manuscript, continuous discussion and guiding the documentation of the project. SM provided design and building of the hive boxes. BP developed and debugged the current hardware version and wrote part of the manuscript. JP wrote hardware and converter code and part of the manuscript. PS as a practical beekeeper he made substantial suggestions of the construction of the hive. TS wrote the capacitance and temperature and humidity sensor driver. AV ran the collaborations with beekeepers and analyzed their data. SW analyzed the labeled social signals. ZW verified ESF signals. All authors contributed to the article and approved the submitted version.

## Conflict of Interest

PS was owner of the company Hape Imkerei GmbH. The remaining authors declare that the research was conducted in the absence of any commercial or financial relationships that could be construed as a potential conflict of interest.

## Footnotes

^1^https://darksky.net/dev
